# Fruit Carbohydrates and Their Impact on the Glycemic Index: A Study of Key Determinants

**DOI:** 10.3390/foods14040646

**Published:** 2025-02-14

**Authors:** Manish Kumar Singh, Sunhee Han, Songhyun Ju, Jyotsna Suresh Ranbhise, Salima Akter, Sung Soo Kim, Insug Kang

**Affiliations:** 1 Department of Biochemistry and Molecular Biology, School of Medicine, Kyung Hee University, Seoul 02447, Republic of Korea; manishbiochem@gmail.com (M.K.S.); sunheehan@khu.ac.kr (S.H.); thdgus8543@khu.ac.kr (S.J.); jogm25@khu.ac.kr (J.S.R.); aktersalima@gmail.com (S.A.); 2 Biomedical Science Institute, Kyung Hee University, Seoul 02447, Republic of Korea; 3 Department of Biomedical Science, Graduate School, Kyung Hee University, Seoul 02447, Republic of Korea

**Keywords:** carbohydrates, chronic diseases, dietary fiber, fruits, glucose, glycemic index

## Abstract

**Background**: Fruits are a convenient and natural source of carbohydrates that can rapidly affect blood sugar levels and the glycemic index (GI). The GI plays a crucial role in the management of chronic diseases, including diabetes, obesity, hyperglycemia, and diet-related illnesses. Despite there being several health benefits linked with consuming fruits, it remains unclear which specific components of fruits are the key determinants that significantly influence the GI. **Methods**: This study retrospectively examined the relationship between different types of carbohydrates and the GI of various fruits to determine their correlation. The fruits’ sugar and fiber contents were identified from available public databases, the U.S. Department of Agriculture (USDA), FooDB, PubMed, and published sources. **Results**: Previously, the GI was determined by the available carbohydrates, which include different types of sugar. In this study, individual hexose sugars, along with the total carbohydrates and dietary fiber, were examined. The results indicated a strong correlation between fructose and the GI, whereas glucose and total glucose did not exhibit such a correlation. The total carbohydrate-to-fiber ratio displayed a stronger correlation (R = 0.57 and *p* > 0.0001) with the GI compared to glucose alone (R = 0.37; *p* = 0.01) or the total glucose (R = 0.45; *p* = 0.0009) with the consideration of fiber, while the scattering of data points around the regression line suggested that factors beyond the total carbohydrate and fiber also contribute to determining the GI. **Conclusions**: This study demonstrated that individual hexose sugars, especially fructose, significantly influence the GI. These findings suggest that the carbohydrate-to-fiber ratio may offer a more accurate and reliable metric for determining the GI than traditional methods. Further research is warranted to investigate the specific contribution of dietary fiber components, fruit texture, micronutrients, vitamins, genetic predispositions, gut microbiota, and the body’s physiological status to gain a deeper understanding of GI regulation.

## 1. Introduction

Fruits are a vital component of the human diet, providing essential nutrients such as water, carbohydrates, dietary fiber, vitamins, and minerals. They are also rich in potentially bioactive compounds known as flavonoids and phytochemicals [1]. Additionally, fruits are high in antioxidants, which help to protect from oxidative stress and promote overall health benefits. These antioxidants interact with various components, such as carbohydrates, lipids, and proteins, to form a complex food matrix [2]. With the rising prevalence of chronic diseases, such as insulin resistance, diabetes, obesity, non-alcoholic fatty liver disease (NAFLD), cardiovascular disease, and cancers, the role of carbohydrates in managing these conditions has gained significance [3,4,5]. Fruits primarily contain different types of hexose sugars, like glucose, fructose, and disaccharides, such as sucrose, leading to concerns about their consumption and impact on metabolic health.

The specific impact of fruit components depends on their bioaccessibility, the bioavailability of bioactive compounds, and their antioxidant activities [6,7]. Many studies have shown that fruit carbohydrates are linked to the etiology of several chronic diseases [8]. The sugars present in fruits can rapidly elevate glucose spikes and, thus, influence the GI. The GI is calculated as the blood glucose response, which is measured as the incremental area under the curve (iAUC) and expressed as a percentage of the AUC after the consumption of a test meal and then divided by the iAUC for a reference meal containing an equivalent amount of carbohydrate (glucose, white bread) [9,10]. Glucose, a monosaccharide, induces a large glycemic response and is often used as the reference food and assigned a GI of 100 [11]. Fruits with a lower GI reduce the prevalence of chronic diseases, as supported by numerous studies, including meta-analyses [12,13].

The risk factors for chronic diseases can be managed based on the established link between the GI and blood glucose levels. The primary factor influencing the GI in foods, especially the carbohydrate portion of a meal, remains uncertain in food materials like cereals, grains, and fruits. Intriguingly, an important question arises: what type of fruit and how large a quantity should be consumed to prevent a certain spike in blood glucose levels? While the GI is typically assessed via the AUC, researchers are not unanimous about the accuracy and reproducibility of this procedure [10,14]. This has led to discussions on the best method for evaluating the GI and identifying the major influencing factors, such as glucose or fructose; the types of starch, including polysaccharides; and soluble or insoluble dietary fibers. Insoluble dietary fiber, including pectin polysaccharides, mainly consists of xyloglucans and cellulose [15]. Polysaccharides in soluble, fermentable dietary fiber have the ability to promote the growth of microbiomes in the colon and contribute to the digestion of food [16,17]. The bioavailable fraction of these fibers and their total content in modulating blood glucose levels are uncertain.

Recent reports have shown that diet-associated chronic diseases are increasing significantly among Americans and pose significant public health concerns [18,19]. The Dietary Guidelines for Americans 2020–2025, issued by the USDA and the U.S. Department of Health and Human Services (HHS), underscore the importance of this topic, with revisions occurring every five years. The guidelines recommend adopting a healthy dietary pattern that includes nutrient-dense foods, such as vegetables, whole fruits, grains, dairy products, proteins, and oils—mainly vegetable oils, seafood, and nuts. Customized recommendations for each age group in this context are demonstrated in the Dietary Guidelines for Americans (DGA) 2020–2025 by Phillip J.A. [18].

Fiber improves postprandial glucose and insulin responses by slowing sugar absorption and insulin release, thereby promoting satiety through delayed hunger and reduced energy intake [20]. Fruits and vegetables are considered as a vital source of nutrients and dietary fiber, and several plant sterols, flavonoids, and antioxidants present in them have been reported to be associated with body weight management and a reduced risk of chronic disease [21,22]. However, there is considerable uncertainty about which specific fruit and how large a quantity provide the most significant protection against high blood glucose and contribute to overall health benefits. In this study, we investigated the relationship between fruits’ GIs, individual hexose sugars, and dietary fiber. Specifically, we investigated the major key determining factor influencing the GI, including the contribution of monosaccharides, such as glucose or fructose, and dietary fiber and their impact on the GI. Finally, the present study concluded that the personalized prescription of fruits for patients with metabolic diseases may play an important role in managing blood sugar levels and improving health outcomes.

## 2. Materials and Methods

### 2.1. The Sources of the Carbohydrate Contents of the Fruits

In this study, a diverse selection of fruits was selected, based on their availability. The selection process considered multiple factors, such as their taste, nutritional value, health benefits, disease prevention potential, and high anti-inflammatory and antioxidant properties. Initially, the fruits were chosen based on whether they are commonly consumed in their ripe and unripe forms, as well as the amount of fiber that exists within them, which promotes digestive health and regulates blood glucose levels. Subsequently, the selection was refined to focus on the fruits that are widely available and most consumed, either in their whole form or as one of their derivatives (e.g., juice, jelly, and jam). Another important criterion was the presence of well-characterized plant compounds that have been analytically studied in the laboratories. Additionally, the selected fruits are linked to various health benefits, such as high antioxidant contents, low sugar levels, and high fiber contents, which support weight management and reduce the risk of chronic disease. Certain fruits with high water contents, such as watermelons, tangerines, tomatoes, and berries, were also included due to their hydrating properties. These fruits help maintain the body’s acid–base balance, neutralize excess acidity, and contribute to overall health and well-being.

The individual components of the fruits, including carbohydrates and dietary fiber, were identified from the USDA database and other authentic databases. The analytical values for the individual components in each fruit were extracted per 100 g of the fruit. The total carbohydrate (the cumulative amount of all types of sugars) and the total sugar (the sum of hexoses such as glucose, fructose, and sucrose) amounts were taken from different data sources. The specific values for glucose, fructose, and sucrose were also obtained from the USDA Food Data Central, FooDB, Korean Food Composition Database (KFCD), European Food and Safety Authority, World Health Organization (WHO) Nutrient Data Portal, Food Standards Australia-New Zealand, PubMed, and open-access food composition databases. Based on their availability and consumption, we selected all the popular fruits, including berries and tropical fruits, for our investigation.

### 2.2. The Sources of the Glycemic Index (GI) and the Glycemic Load (GL) of the Fruits

The GI and GL values of each fruit were obtained from publicly available online sources, such as the NHS Foundation Trust, which contains values tested after publication. Additional sources for some of the fruits’ data included MyPlate.gov (USDA) accessed on 2 August 2024, GestationalDiabetic.com accessed on 3 August 2024, NourishedByScience.com accessed on 21 September, the online Glycemic Index Database (www.gilisting.com) accessed on 5 August 2024, and the official GI website and databases (www.glycemicindex.com) accessed on 24 July 2024.

### 2.3. Statistical Analyses

All the statistical analyses were conducted using Excel and GraphPad Prism (version 5.0). The Pearson’s linear correlation test was employed to assess the relationship between the dietary GI, GL, and individual sugar contents. The correlation coefficient (R) and regression line were determined based on the best-fit values for the slope and intercept. The correlation strength was classified as weak for statistically significant R values of below 0.4, moderate for R values between 0.4 and 0.6, and strong for R values above 0.6. The assigned values for the GI and carbohydrates were calculated using biostatistical methods, and the results were expressed as the mean ± SD. The *p*-values were determined using a two-tailed test and a one-way ANOVA with a 95% confidence interval. The statistical significance was set at *p* < 0.05.

## 3. Results

In this study, the primary objective was to investigate the effect of available carbohydrates, especially hexoses, such as glucose and fructose, and dietary fiber, on the GI of various fruits. We categorized the fruits into two main groups: (1) the fruits whose components have been well characterized and documented and (2) the most popular fruits available worldwide (marked with an asterisk (*) in Table 1). The available carbohydrate content of each fruit was calculated as the ratio of individual hexose sugar types (g) to dietary fiber (g). Fruit carbohydrates include various forms of sugars, such as monosaccharides (glucose, fructose, and galactose), disaccharides (sucrose, maltose, and lactose), and polysaccharides (glycogen and starch). Sucrose is a disaccharide that is abundant in fruits and is readily hydrolyzed into equal parts of glucose and fructose. As a result, the total glucose content includes both free glucose and the portion of the glucose obtained from sucrose. Similarly, the total fructose content consists of free fructose along with the fructose derived from sucrose. The total sugar content represents the combined amounts of glucose, fructose, and sucrose, while the total carbohydrate content encompasses all these sugar forms analyzed. The resulting carbohydrate-to-dietary fiber (g) ratios are presented in Table 2. Using individual hexose sugar-to-fiber ratios provides more informative insights than taking the absolute difference between the total carbohydrates and fiber. This approach offers a clearer understanding of how dietary fiber contributes to the total carbohydrate content and the different hexoses, which may influence digestion, glucose absorption, glycemic response, and overall health benefits. Unlike the absolute differences, this ratio sheds light on the effectiveness of fiber in controlling the metabolic effects of individual hexose sugars in a particular fruit. This metric allows us to better assess the physiological relevance of individual hexoses in fruits and helps to identify fruits with a more favorable hexose sugar-to-fiber ratio for managing the glycemic response.

**Figure 1:**
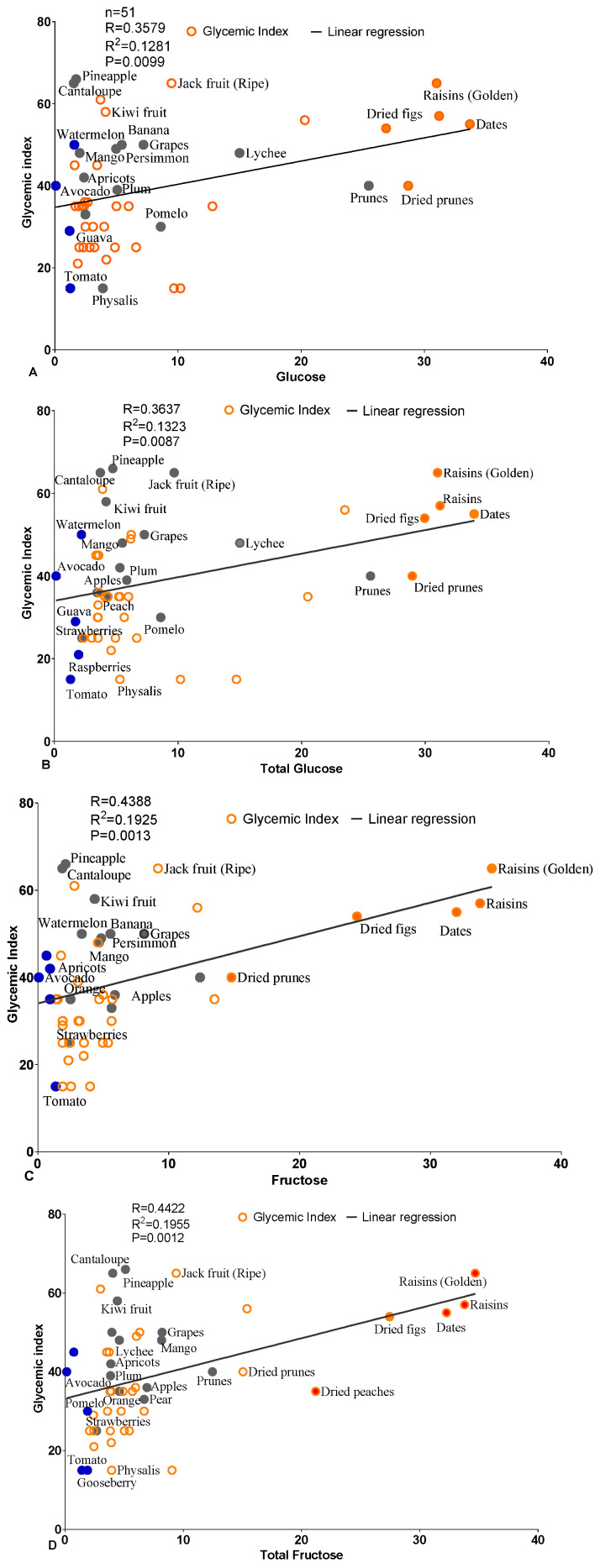

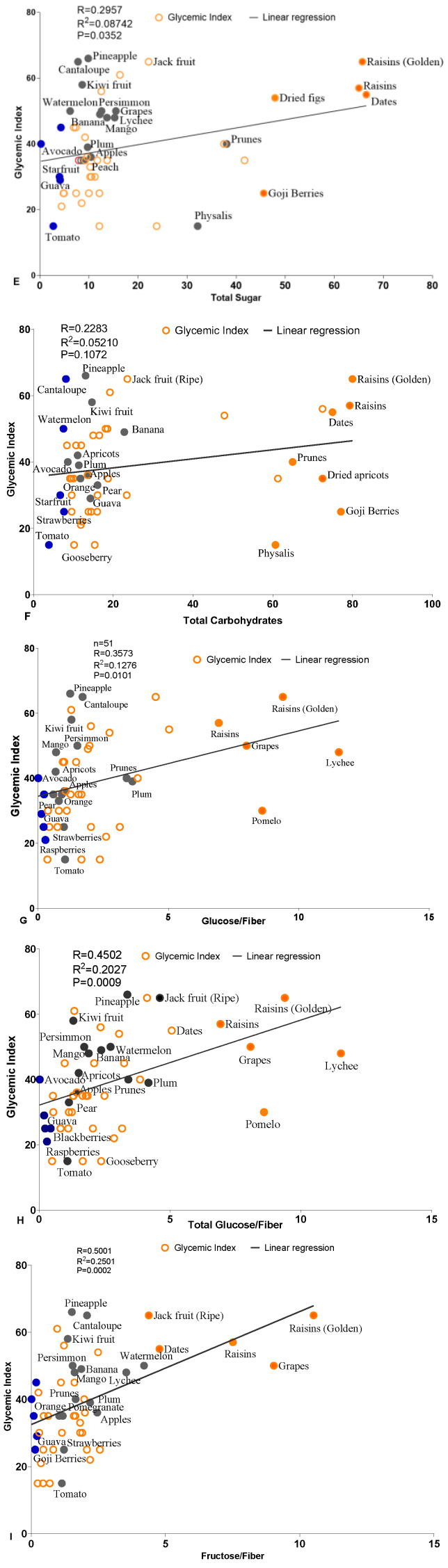

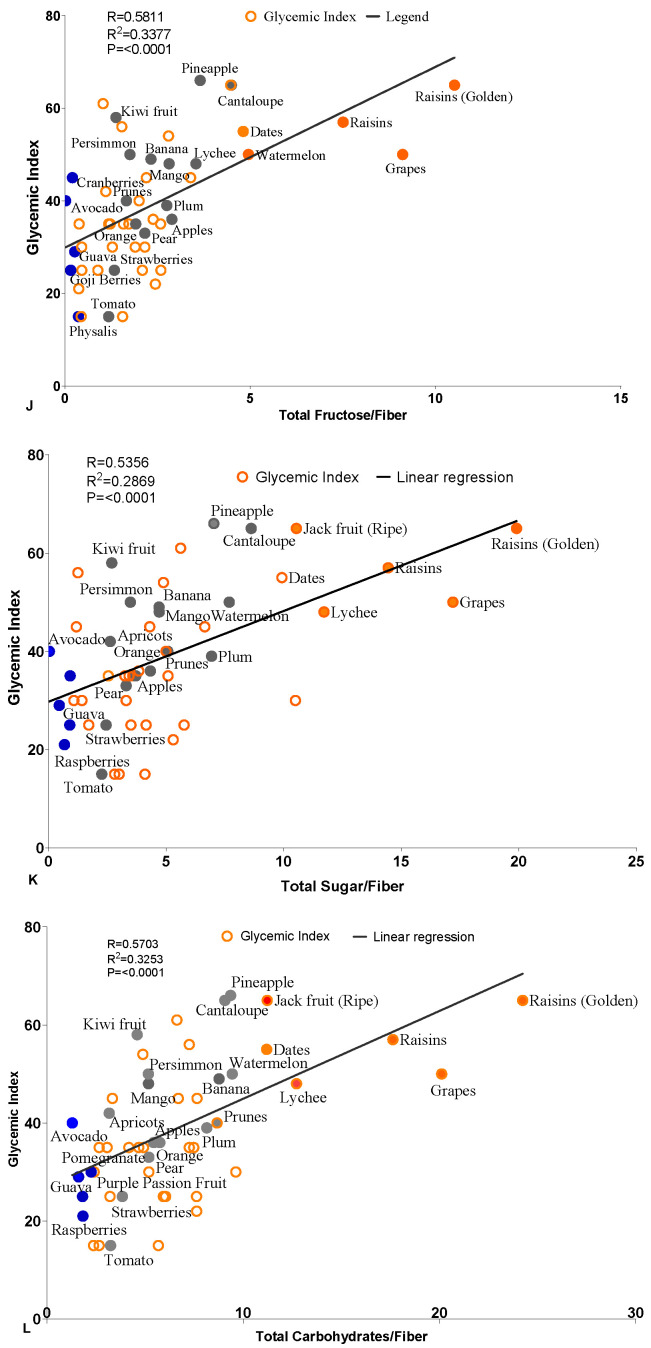
(**A**–**F**): The correlation plot illustrates the relationship between the glycemic index (GI) and the carbohydrate content of various fruits (n = 51). The graphs display the correlation between individual hexose sugar and the GI values. Each data point represents an individual fruit, with the GI values plotted on the *Y*-axis and the sugar contents on the *X*-axis. The solid line indicates the regression curve, showing the trend between the sugar levels and the GIs across the fruit samples. Blue-filled circle: the fruits with the least sugar; orange-bordered circles: the fruits with a medium sugar content; orange-filled circles: the fruits with the highest sugar; grey-filled circles: the most popular fruits. (**G**–**L**): The correlation plot illustrates the relationship between the glycemic index (GI) and the sugar-to-fiber ratio in various fruits (n = 51). Each data point represents an individual fruit, with the GI values plotted on the *Y*-axis and the sugar-to-fiber contents on the *X*-axis. A linear regression line is included to show the relationship between the sugar levels in different fruits and their GI. The graph reveals a trend between the sugar content and the GI across the fruit samples. The *p*-value was calculated using a two-tailed test. Blue-filled circles: the fruits with the lowest sugar-to-fiber ratio; orange-bordered circles: the fruits with a medium sugar-to-fiber ratio; orange-filled circles: the fruits with the maximum sugar-to-fiber ratio; grey-filled circles: the most popular fruits.

The correlation between different hexose contents and the GI in a variety of fruits (n = 51): Our results indicated that glucose, fructose, and total sugar had a strong correlation with the GI. Notably, the total carbohydrates displayed an insignificant correlation, showing lower R or *p*-values (R = 0.22; *p* = 0.1072), while other hexose sugar forms displayed higher R and *p*-values (Table 3). All the data points were scattered across the *X-* and *Y*-axes, with some points located close to the regression line (Figure 1A–F).

Next, the correlation between the different hexose sugar-to-fiber ratios and the GI: The analysis revealed a more significant correlation with the GI. The data analysis indicated that the fruits with lower sugar contents had a stronger correlation with the GI and were located closer to the regression line and the *Y*-axis. In contrast, the fruits with higher GIs and high sugar-to-fiber ratios were scattered far from the regression line. The total carbohydrate-to-fiber ratio displayed the highest significant R and *p*-values (R = 0.57; *p* < 0.0001), followed by the total fructose and the total sugar. Unexpectedly, glucose alone and the total glucose displayed weak correlations and insignificant R values (R = 0.35) and *p*-value (*p* = 0.45); the data points were also scattered away from the regression line (Figure 1G–L; Table 4).

To evaluate the practical relevance of the findings, a specific group of widely available and commonly consumed fruits was selected for further analysis. These fruits were chosen based on their consumption, global popularity, nutritional value, diverse textures, and high production rates. Given their widespread consumption, it was essential to assess their impact on the GI. The results displayed strong correlations between both the total carbohydrate and the total sugar and the GI, while glucose and fructose did not show strong correlations with the GI. Notably, the results demonstrated significant R and *p*-values for the total carbohydrate and the total sugar (R = 0.413 and R = 0.486; *p* = 0.02 and *p* = 0.0087, respectively). In contrast, glucose alone, the total glucose, fructose, and the total fructose did not yield significant R or *p*-values (Figure S1A–F; Table S1A). Further, these fruits were also analyzed for their carbohydrate-to-fiber ratio. The data displayed similar trends with the GI. Glucose alone and the total glucose over fiber ratio did not reach a significant level (Figure S1G–L), and their corresponding R and *p*-values were insignificant (Table S1B). On the other hand, the ratios of the total carbohydrate, total sugar, and total fructose to fiber were significant, with corresponding significant R and *p*-values (R = 0.46, R = 0.44, and R = 0.44 and *p* = 0.011, *p* = 0.014, and *p* = 0.014, respectively). Although the data points were scattered around the regression line, the total carbohydrate-to-fiber ratio exhibited the strongest correlation and was located closest to the regression line.

**Figure 2:**
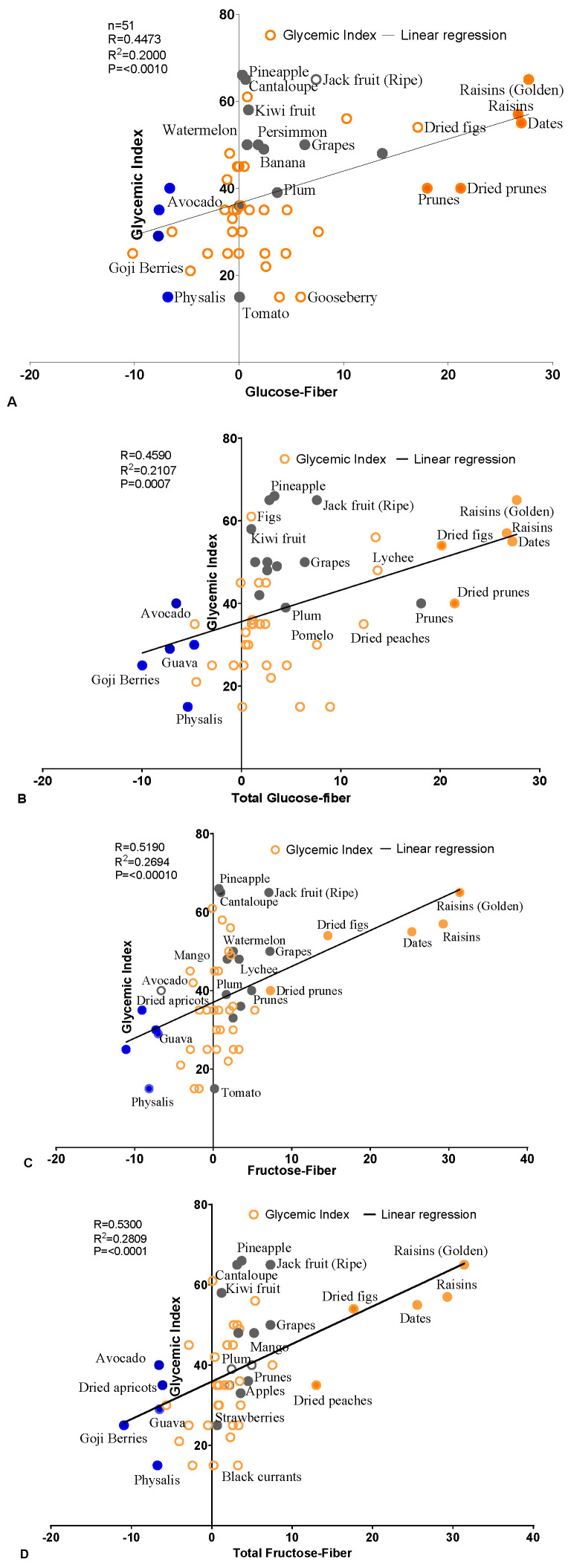

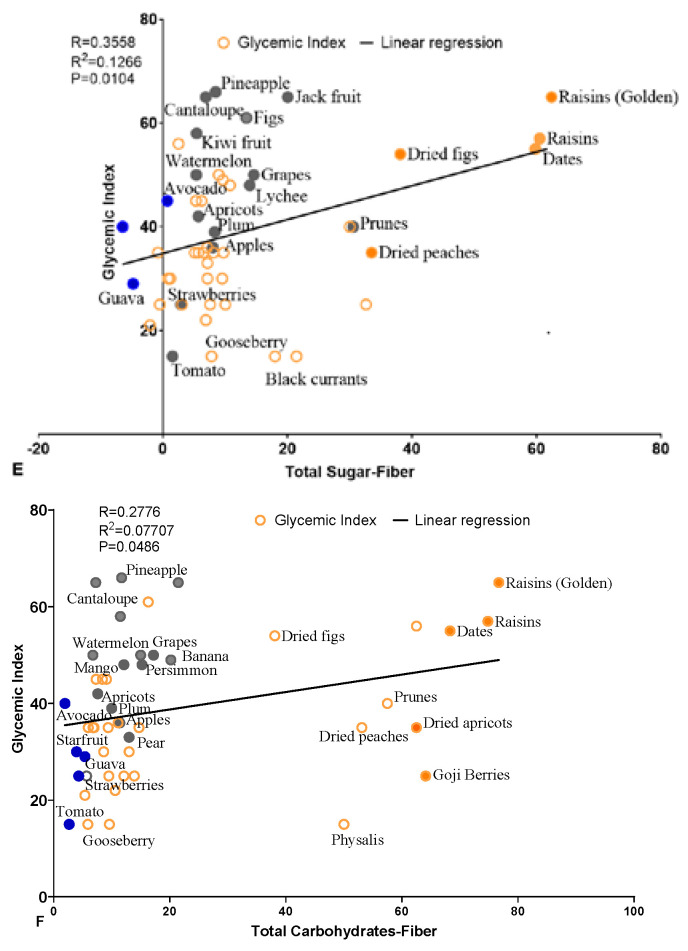
(**A**–**F**): The correlation plot illustrates the relationship between the glycemic index (GI) and the carbohydrates minus the fiber in various fruits (n = 51). Each data point represents an individual fruit, with the GI values displayed on the *Y*-axis and the hexose sugar minus fiber content on the *X*-axis. A linear regression line is included to show the relationship between the sugar levels in different fruits and their GI. The graph illustrates the trend between the sugar content and the GI across the different fruits. The *p*-value was calculated using a two-tailed test. **Blue-filled circles:** fruits with low sugar; **orange-bordered circles**: fruits with medium sugar; orange-filled circles: fruits with high sugar; **grey-filled circles**: most popular fruits.

The above findings were compared with the conventional method used to calculate the GI based on available carbohydrates (g). The available carbohydrates were measured by subtracting the total carbohydrate content from the dietary fiber, using the formula (available carbohydrate (g) = total carbohydrate (g) − dietary fiber (g)) [23]. Surprisingly, the fruits with low total carbohydrate contents relative to their dietary fiber contents showed resultant low available carbohydrates (Table 5). However, the fruits with lower glucose or fructose contents than fiber showed resultant negative values, like avocado, guava, nectarine, peach, and mango. The correlation between the available carbohydrate contents and the GIs of the fruits were examined separately for each group. The results showed that many data points remained scattered along the *X-* and *Y*-axes, while few points aligned close to the regression line, due to the low content of carbohydrates in these fruits. Moreover, the correlation coefficient R and *p*-values were lower compared to the individual hexose sugar-to-fiber ratio (Figure 2A–F, Table 6). Additionally, another group, categorized as the most popular and widely available fruits, contained a smaller number of fruits (n = 27). The results exhibited an insignificant correlation between the carbohydrate content and the GI across all the types of hexose sugars, except for the total fructose. The total fructose exhibited a weak, but statistically significant, correlation with the GI, showing R = 0.3855 and *p* = 0.0389 values (Figure S2A–F; Table S2A).

To improve the clarity and integrity of the above results, some fruits were located far from the regression line, as they were considered outliers and had very high individual hexose sugar contents. The total carbohydrate alone displayed a weak correlation with the GI, with insignificant R and *p*-values (Figure S3A–F, Table S3A). In contrast, a notable improvement was observed with the individual hexose sugar-to-fiber ratios, which displayed higher R and *p*-values across all the types of hexose sugar (Figure S3G–L, Table S3B). Specifically, the total carbohydrate, fructose, and total sugar-to-fiber ratios displayed higher R and *p*-values (ranging from R = 0.57 to R = 0.47 and *p* < 0.0001), while glucose had lower values that were surprising (R = 0.16 and *p* = 0.28). In addition, the data points in all the categories were located close to the regression line, which showed significant improvements regarding their correlation with the GI.

The above findings were also examined with a smaller subset of fruits (n = 27), as mentioned above. The results showed variations from the above findings. The correlation co-efficient between the carbohydrate content and the GI showed more differences across the sugar content and the GI. The results displayed reductions in R and *p*-values, whereas the total carbohydrate, total sugar, and total fructose displayed significant correlations with the GI, with R = 0.41, R = 0.38, and R = 0.37 and *p* = 0.02, *p* = 0.04 and *p* = 0.04 values, respectively. In contrast, glucose alone, the total glucose, and fructose displayed insignificant correlations with the GI (Figure S4A–F; Table S4A). The data points were more scattered along the *X-* and *Y*-axes and were located farther away from the regression line. However, individual hexose sugar-to-fiber ratio showed improved results, with strong correlations with the total carbohydrate (R = 0.51), total sugar (R = 0.47), total fructose (R = 0.51), and total glucose (R = 0.50), as well as *p*-values (*p* = 0.0053, *p* = 0.014, *p* = 0.008, and *p* = 0.0048, respectively) (Figure S4G–L, Table S4B).

Some studies have indicated that the glycemic load (GL) is an important determinant of an increased risk of diabetes and insulin release. To investigate this, a Pearson’s linear correlation analysis was conducted to explore the relation between the fruits’ sugars, especially hexoses, and the GL. The results revealed a pattern similar to that observed between the carbohydrate content and the GI, as well as between the carbohydrate-to-fiber ratio and the GI. A significant correlation was found between individual sugars and the GI across all the fruits (Figure S5A–F; Table S5A). Among these sugars, fructose exhibited the strongest correlation, with higher R and *p*-values compared to glucose and the total glucose. Subsequently, the correlation between the different hexose sugar-to-fiber ratios and the GL followed a similar trend. The GL was positively correlated with the total carbohydrates, fructose and total fructose, while glucose and the total glucose displayed a moderate correlation, with lower R and *p*-values. Notably, the fruits with lower sugar contents showed a stronger correlation with the GL, clustering closer to the regression line and the Y-axis. In contrast, the high-sugar fruits demonstrated a weaker correlation and were more widely scattered on the graph (Figure S5G–L; Table S5B). A similar analysis was also conducted with another group of fruits (n = 29), and the corresponding R and *p*-values were presented (Table S6A,B). The carbohydrates showed a significant association with the GL, while the carbohydrate-to-fiber ratio and the GL did not demonstrate a significant relationship. The R and *p*-values in this analysis were lower compared to those from the analysis based on the larger number of fruits.

## 4. Discussion

The GI plays a vital role in maintaining physiological homeostasis, especially in managing chronic diseases. Studies have shown that the proportion of carbohydrates and fiber is crucial for assessing food quality. A diet rich in fiber and low in carbohydrate quantity reduces the risk of chronic diseases [23]. The GI is primarily determined by the available carbohydrate content in food, which is the remaining carbohydrate amount after subtracting dietary fiber from the total carbohydrates, then comparing the AUC with an equivalent amount of a reference hexose sugar, such as glucose [24]. In this study, we focused on fruit-derived carbohydrates, mainly monosaccharides, such as glucose and fructose, as well as the total sugar and carbohydrates and their ratio to dietary fiber. Based on the fruits’ contents, specifically their available carbohydrates and fiber, we proposed that the carbohydrate-to-fiber ratio serves as a more reliable metric for estimating the GI than the difference between carbohydrates and fiber. Our findings showed that this ratio across various fruits is more positively associated with the GI. Surprisingly, our analysis displayed neither glucose nor the total glucose, and their ratios to fiber did not show a strong correlation with the GI. However, fructose, as well as the total carbohydrates and their ratio to dietary fiber, displayed a stronger correlation with the GI than glucose. Notably, in the regression analysis for fructose and the total fructose, most of the fruits were aligned near the regression line, with the Y-axis under-scoring their significant association with the GI. Higher R values for fructose and the total fructose, alongside significant *p*-values, suggest that fructose might be a more sensitive determinant of the GI. Although the underlying cellular mechanism remains to be fully elucidated, our findings highlight the potential role of fructose in influencing the GI.

The GL is calculated as the product of the GI and the total available carbohydrates content in a given portion of food (GL= GI × available carbohydrate/serving amount of food). It reflects the overall impact of carbohydrate intake on blood glucose levels. From a health perspective, GL has been found to have a stronger association with plasma lipid levels than the insulin response or glycemic fluctuations [25,26]. However, relatively few studies have explored this aspect in detail. In this analysis, the GL showed a stronger correlation with fructose compared to other hexose sugars, such as glucose. This suggests that fructose might serve as an alternative to mitigate elevated glucose levels in individuals with T2DM and insulin resistance. Conversely, extensive research has also linked fructose consumption to an increased risk of obesity, diabetes, and cardiovascular diseases, making its role in metabolic health a topic of ongoing debate [27]. Therefore, accurately quantifying the carbohydrate compositions in fruits and assessing their effects on the GL could provide valuable insights into postprandial glucose regulation. Understanding these relationships may help in designing dietary strategies that optimize the glycemic response, which is particularly beneficial for individuals with diabetes and obesity.

Fruits are a natural source of dietary fiber and contribute to the digestion and absorption of carbohydrates. The proportion of soluble and insoluble fibers varies considerably across the fruits, depending on the analytical method used for quantitation [28,29]. Soluble dietary fiber binds effectively to glucose and acts as a physical barrier that slows glucose absorption, thereby having a major effect on postprandial blood glucose levels [28]. This synergistic action makes total dietary fiber a valuable food ingredient in regulating blood glucose levels and the GI [30]. Fructose is derived from various carbohydrate sources, like monosaccharides, disaccharides (e.g., sucrose), and oligosaccharides, in fruits. Fructose needs less enzymatic digestion before being transported into the enterocyte and the hepatic portal vein via GLUT2 and GLUT5 transporters [31]. Additionally, fructose is often used as an alternative sweetener in various food products and beverages, raising concerns about its role in the increasing rates of obesity, diabetes, and related cardiometabolic complications. Animal studies have shown that controlled fructose consumption, when substituted isocalorically for other carbohydrates at low-to-moderate doses (catalytic doses of ≤10 g per meal) comparable to the levels naturally found in fruits, does not appear to have negative health effects [32]. In contrast, the excessive intake of fructose (≥100 g/day) has been linked to metabolic disorders in humans [32,33]. Despite these findings, the evidence on this topic remains limited, and further research is necessary to better understand the impact of fructose from fruit sources on overall health.

While numerous studies have explored the role of dietary fiber in glucose metabolism, limited evidence exists regarding how dietary fibers influence the absorption and assimilation of hexose sugars, such as fructose and galactose, in a meal. This gap in the knowledge highlights the need for further investigation into the broader effects of dietary fibers on the metabolism of hexose sugars beyond glucose. Recent studies have reported that soluble fiber, in particular, affects the glycemic response through its interaction with gut microbes [34,35]. Fibers are metabolized by gut microbes to produce various metabolites, such as short-chain fatty acids (SCFAs), like butyrate, acetate, and propionate, which support colon bacteria for growth and proliferation, while the remaining undigested fibers increase the fecal bulk. Comprehensive data on fiber types, the fraction of starch, and their interaction with glucose and other carbohydrates across all fruits are limited, and, thus, more detailed information is warranted. We suggest that future research incorporating these variables may provide greater insight into how individual hexose sugars affect the GI.

In addition to dietary fiber, various factors such as micronutrients, fruit texture (e.g., firmness, ripeness), genetic factors, and non-genetic factors, including diet, exercise, and environmental conditions, also contribute to chronic disease progression through epigenetic modifications [36]. For instance, aging leads to changes in DNA methylation, which may increase the prevalence of such diseases. Recent studies on mice have shown that the maternal consumption of a Western diet containing refined carbohydrates, saturated fats, and low fiber can result in epigenetic modifications, such as the hypermethylation of the ApoB gene, predisposing male offspring to NAFLD [37]. In this study, there are many fruits with comparable fiber contents that impact on the absorption of sugars. Fruits high in fiber can help compensate for a low fiber intake in the diet, thereby increasing nutrient absorption, neutralizing free radicals, and reducing gene modifications, thus promoting overall health benefits. However, the exact role of dietary fiber in modifying genes and reducing the chronic disease risk remains under-explored. Alterations in dietary patterns in genetically predisposed individuals may affect specific clinical outcomes, such as metabolic function and the inflammatory response. Personalized nutrition therapy, involving adjustments to dietary components, such as increasing dietary fiber intake and reducing refined carbohydrates, has the potential to address these genetic and non-genetic factors, with environmental conditions helping to reduce the harmful effects of certain dietary components.

Furthermore, a high-sugar and low-fiber diet significantly influences hormone signaling. For instance, a Western-style diet can increase cortisol release, leading to cellular responses to metabolic signals [38,39,40,41]. This response is influenced by an individual’s physiological status, including systemic inflammation, visceral fat accumulation, the standard of living, and the level of glucose intolerance [42,43]. Western diets are characterized by highly refined sugars, animal-based proteins, and low plant-based fibers. A high dietary fat intake generates more reactive oxygen species (ROS) in mitochondria, impairing insulin sensitivity and exacerbating metabolic dysfunction [44]. Increased ROS are considered a major source of hyperglycemia-induced complications in diabetes, contributing to insulin resistance, dyslipidemia, and β-cell dysfunction, which leads to impaired glucose tolerance [45]. In contrast, fruits and vegetables are rich in phytochemicals that act as antioxidants, scavenging free radicals and reducing the risk of chronic diseases [46]. A balanced diet rich in fruits and vegetables is, therefore, essential for counteracting the harmful effects of metabolic dysfunction and supporting overall metabolic benefits [47,48,49]. This analysis listed various fruits with their nutritional values, including avocado, guava, raspberry, and blackberry, which are low in carbohydrates and high in fiber. Various berries, such as cranberries, strawberries, raspberries, blackberries, gooseberries, and goji berries, exhibited a low fiber ratio with fructose and were located near the regression line, indicating a strong correlation with the GI. Another study also demonstrated that consuming mixed berries with white or rye bread reduces the postprandial insulin response, thus impacting the GI [50]. These findings indicate that incorporating such fruits into a Western diet can improve insulin secretion and the glycemic response, thus minimizing negative impacts.

Continuous glucose monitoring (CGM) is an effective tool for monitoring blood glucose levels in type-1 or type-2 diabetes. CGM data can be useful in reducing glucose fluctuations and minimizing the risk of hypoglycemia [51,52,53,54]. CGM systems include real-time monitoring (RT-CGM) and flash or intermittently scanned continuous glucose monitoring (FGM or isCGM), which measure interstitial glucose levels via a subcutaneous glucose sensor [55]. CGM data aid in accurate diagnoses, treatment strategies, and the assessment of glucose tolerance and insulin resistance [56]. CGM can assist in understanding how carbohydrates, particularly hexose sugars, such as glucose or fructose, affect blood glucose levels two hours after food intake. The findings in this study indicated that fruits like avocado, strawberries, and guava have low total carbohydrate contents and displayed low carbohydrate-to-fiber ratios, thus closely correlating with the GI, and were located near the Y-axis. Conversely, grapes, lychee, and jackfruit have more carbohydrates than dietary fiber. Thus, a high ratio exhibited a weaker correlation with the GI and was located far from the Y-axis. Therefore, their ratio provides more precision to determine the available carbohydrate that influences blood glucose levels. The regular monitoring of blood glucose levels using CGM after consuming these fruits for certain periods may aid in the size adjustment for high-sugar fruits based on individual HbA1c levels and glucose tolerance.

Other factors, such as the fruit sugar content, stress, exercise, sleep, and physical condition, can also impact blood glucose levels. Monitoring blood glucose levels after eating a certain amount of fruit can identify those with the least impact, allowing for better dietary choices. Although these devices offer better accuracy and utility, high costs and limited access are major challenges. Broadly, RT-CGM interventions lasting 24 weeks or longer have proven to be very effective in reducing hemoglobin A1c (HbA1c) levels over specified periods [57]. Therefore, the longer monitoring of RT-CGM data is more effective in reducing blood sugar levels [58]. Nevertheless, the lack of structured education on the use of CGM limits its potential benefits [56]. In addition to clinical applications, CGM monitoring can also assist individuals in optimizing their fruit choices and portion sizes based on glucose responses and HbA1c levels, presenting a cost-effective strategy for glycemic regulation. Further exploration of these variables is necessary for a comprehensive understanding.

## 5. Conclusions

Fruit intake may affect the GI and potentially increase the risk of developing chronic diseases [59,60]. Several epidemiological studies have explored the relationship between fruit intake and risk of T2D; however, those findings have been inconclusive [61,62]. The GI ranks carbohydrate-containing foods based on their ability to raise blood glucose levels, with an equal quantity of a reference sugar, such as glucose or white bread [63]. Foods high in carbohydrates and low in fiber typically have a high GI, leading to a rapid increase in blood sugar levels. In contrast, foods with a lower ratio suggest a lower GI and have less impact on blood glucose levels. Using the ratio of carbohydrates and fiber provides a more significant correlation with the GI. Since fruits vary widely in size and composition, these ratios help normalize those variations, making it easier to compare different types and serving sizes of fruit. Furthermore, it would be valuable to evaluate the other components of fruits to better understand their effects on glucose homeostasis.

For individuals with diabetes or who are controlling weight, knowing the carbohydrate-to-fiber ratio, rather than relying only on the glucose or fiber content, is important for selecting fruits that meet their nutritional requirements. Focusing on these ratios provides a more comprehensive view of the nutritional and functional interplay between carbohydrates and fiber, which is particularly important when examining health benefits or recommending dietary changes. In addition, individual physiological conditions should be considered when creating personalized fruit recommendations for the prevention and management of blood glucose levels. Further research is necessary to validate these findings across diverse populations and to enhance our understanding of how fruit composition interacts with glycemic regulation.

### Limitations

To ensure the accuracy and relevance of these findings, further confirmations through in vivo or clinical trials are required. This study did not include other factors, such as the gut microbiome, fruit texture, hormonal secretions, and genetic factors, that may be involved in determining the GI. Future investigation into these factors will provide more information. The use of CGM and the monitoring of blood sugar levels after the ingestion of meals with high and low GIs and high and low fiber contents may also impact such calculations. Such personalized guidance can greatly aid in managing the GI effectively, ultimately reducing the risk of chronic diseases and promoting overall health benefits.

## Figures and Tables

**Table 1:** The composition of sugar (g), expressed per 100 g serving, ranked in the ascending order of the glucose content, along with the glycemic index (GI), glycemic load (GL), and dietary fiber contents of various fruits.

S. No.	Fruit Name	Glucose	Total Glucose	Fructose	Total Fructose	Sucrose	Total Sugar	Total Carbohydrate	Glycemic Index	Glycemic Load	Fiber
1	Avocado *	0.08	0.14	0.08	0.14	0.06	0.22	8.64	40	3	6.7
2	Guava *	1.2	1.7	1.9	2.4	1	4.1	14.3	29	3	8.9
3	Tomato *	1.25	1.3	1.37	1.42	0.1	2.72	3.89	15	1.1	1.2
4	Cantaloupe *	1.54	3.715	1.87	4.045	4.35	7.76	8.16	65	4	0.9
5	Watermelon *	1.58	2.185	3.36	3.965	1.21	6.15	7.55	50	5.6	0.8
6	Grapefruit (Pink)	1.61	3.365	1.77	3.525	3.51	6.89	10.7	45	5.4	1.6
7	Grapefruit (White)	1.61	3.575	1.77	3.735	3.93	7.31	8.41	45	8.1	1.1
8	Nectarine	1.67	4.085	1.39	3.805	4.83	7.89	9.18	35	4.1	2.2
9	Pineapple *	1.73	4.725	2.12	5.115	5.99	9.84	13.1	66	8.6	1.4
10	Raspberry	1.86	1.96	2.35	2.45	0.2	4.41	11.9	21	3	6.5
11	Pomelo	1.9	3.3	1.9	3.3	2.8	6.6	9.62	30	3	1
12	Peach *	1.95	4.33	1.53	3.91	4.76	8.39	10.1	35	4	3.3
13	Strawberry *	1.99	2.225	2.44	2.675	0.47	4.9	7.68	25	1.9	2
14	Mango *	2.01	5.495	4.68	8.165	6.97	13.66	15	48	8.4	2.9
15	Gooseberry	2.15	3.08	1.87	2.8	1.86	5.88	11.34	15	1.8	4.3
16	Orange *	2.2	4.3	2.5	4.6	4.2	8.9	11.8	35	4.1	2.4
17	Blackberry *	2.31	2.345	2.4	2.435	0.07	4.78	9.61	25	2.5	5.3
18	Apricot *	2.37	5.305	0.94	3.875	5.87	9.18	11.1	42	12.9	3.5
19	Dried Apricot	2.37	5.305	0.94	3.875	5.87	9.18	72.5	35	21.2	10
20	Apple *	2.43	3.465	5.9	6.935	2.07	10.4	13.8	36	5.04	2.4
21	Pear *	2.48	3.535	5.64	6.695	2.11	10.23	16.1	33	4.7	3.1
22	Pear (Bosc)	2.48	3.535	5.64	6.695	2.11	10.23	16.1	30	4.7	3.1
23	Green Apple	2.66	3.625	5	5.965	1.93	9.59	13.6	36	5	2.5
24	Goji Berry	2.84	3.02	1.9	2.08	0.36	45.6	77.1	25	13.3	13
25	Starfruit	3.1	3.5	3.2	3.6	0.8	3.98	6.73	30	1.2	2.8
26	Red Currant *	3.22	3.525	3.53	3.835	0.61	7.36	13.8	25	1.9	4.3
27	Cranberry *	3.44	3.52	0.67	0.75	0.16	4.27	12	45	5.5	3.6
28	Fig *	3.7	3.9	2.8	3	0.4	16.3	19.2	61	28.9	2.9
29	Physalis	3.9	5.305	2.54	3.945	2.81	32.14	60.71	15	0.6	10.7
30	Purple Passion	4	5.65	3.1	4.75	3.3	11.2	23.4	30	6.9	10.4
31	Kiwi (Fruit) *	4.11	4.185	4.35	4.425	0.15	8.61	14.7	58	3	3.2
32	Cherries (Sour)	4.18	4.58	3.51	3.91	0.8	8.49	12.2	22	2.5	1.6
33	Blueberry *	4.88	4.935	4.97	5.025	0.11	9.96	14.5	25	2	2.4
34	Banana *	4.98	6.175	4.85	6.045	2.39	12.22	22.8	49	10.1	2.6
35	Pomegranate *	5	5.2	4.7	4.9	0.4	13.7	18.7	35	6.7	4
36	Plum *	5.07	5.855	3.07	3.855	1.57	9.71	11.4	39	3.9	1.4
37	Persimmon *	5.44	6.21	5.56	6.33	1.54	12.54	18.6	50	7.7	3.6
38	Prickly Pear	6	6	5.7	5.7	0	11.7	9.57	35	3.4	3.6
40	Sweet Cherry *	6.59	6.665	5.37	5.445	0.15	12.11	16	25	0.1	2.1
41	Grape *	7.2	7.275	8.13	8.205	0.15	15.48	18.1	50	9.6	0.9
42	Jackfruit * (Ripe)	9.48	9.69	9.19	9.4	0.42	22.14	23.57	65	17.6	2.1
43	Blackcurrant	9.68	14.73	4	9.05	10.1	23.78	15.4	15	1.1	5.8
44	Dried Peach	12.8	20.5	13.5	21.2	15.4	41.7	61.3	35	20.2	8.2
45	Lychee *	15	15	4.6	4.6	0	15.23	16.53	48	1	1.3
46	Dried apricot	20.3	23.5	12.2	15.4	6.4	12.5	72.5	56	3.8	10
47	Prune *	25.5	25.575	12.4	12.475	0.15	38.05	65	40	25.6	7.5
48	Dried Fig	26.9	29.95	24.4	27.45	6.1	47.9	47.9	54	28.9	9.8
49	Dried Prune	28.7	28.95	14.8	15.05	0.5	37.5	65	40	25.6	7.5
50	Raisin (Golden)	31	31	34.7	34.7	0	65.7	80	65	46.8	3.3
51	Raisin	31.2	31.2	33.8	33.8	0	65	79.32	57	51.5	4.5
52	Date (Fruit)	33.7	33.965	32	32.265	0.53	66.5	75	55	8	6.7

Note: The total glucose is calculated as the sum of the free glucose and half the sucrose amount; the total fructose is the sum of the free fructose and half the sucrose amount; and the total sugar is the sum of the glucose, fructose, and sucrose. The data presented are mean values derived from published sources or provided as a single value from a database. The fruits marked with an asterisk (*) represent a distinct group (the most popular fruits) used for analysis.

**Table 2:** Ratio of carbohydrate content-to-fiber in various fruits.

S. No.	Fruits Name	Ratio of Glucose and Fiber	Ratio of Total Glucose and Fiber	Ratio of Fructose and Fiber	Ratio of Total Fructose and Fiber	Ratio of Total Sugar and Fiber	Ratio of Total Carbohydrate and Fiber
1	Avocado *	0.01194	0.0209	0.01194	0.0209	0.03284	1.28955
2	Guava *	0.13483	0.19101	0.21348	0.26966	0.46067	1.60674
3	Tomato *	1.04167	1.08333	1.14167	1.18333	2.26667	3.24167
4	Cantaloupe *	1.71111	4.12778	2.07778	4.49444	8.62222	9.06667
5	Watermelon *	1.975	2.73125	4.2	4.95625	7.6875	9.4375
6	Grapefruit (Pink)	1.00625	2.10313	1.10625	2.20313	4.30625	6.6875
7	Grapefruit (White)	1.46364	3.25	1.60909	3.39545	6.64545	7.64545
8	Nectarine	0.75909	1.85682	0.63182	1.72955	3.58636	4.17273
9	Pineapple *	1.23571	3.375	1.51429	3.65357	7.02857	9.35714
10	Raspberry	0.28615	0.30154	0.36154	0.37692	0.67846	1.83077
11	Peach *	0.59091	1.31212	0.46364	1.18485	2.54242	3.06061
12	Strawberry *	0.995	1.1125	1.22	1.3375	2.45	3.84
13	Mango *	0.6931	1.89483	1.61379	2.81552	4.71034	5.17241
14	Orange *	0.91667	1.79167	1.04167	1.91667	3.70833	4.91667
15	Blackberry *	0.43585	0.44245	0.45283	0.45943	0.90189	1.81321
16	Apricot *	0.67714	1.51571	0.26857	1.10714	2.62286	3.17143
17	Dried Apricot	0.237	0.5305	0.094	0.3875	0.918	7.25
18	Apple *	1.0125	1.44375	2.45833	2.88958	4.33333	5.75
19	Pear *	0.8	1.14032	1.81935	2.15968	3.3	5.19355
20	Pear (Bosc)	0.8	1.14032	1.81935	2.15968	3.3	5.19355
21	Green Apple	1.064	1.45	2	2.386	3.836	5.44
22	Goji Berry	0.21846	0.23231	0.14615	0.16	3.50769	5.93077
23	Starfruit	1.10714	1.25	1.14286	1.28571	1.42143	2.40357
24	Red Currant *	0.74884	0.81977	0.82093	0.89186	1.71163	3.2093
25	Cranberry *	0.95556	0.97778	0.18611	0.20833	1.18611	3.33333
26	Fig *	1.27586	1.34483	0.96552	1.03448	5.62069	6.62069
27	Physalis	0.36449	0.49579	0.23738	0.36869	3.00374	5.67383
28	Purple Passion	0.38462	0.54327	0.29808	0.45673	1.07692	2.25
29	Kiwi (Fruit) *	1.28438	1.30781	1.35938	1.38281	2.69063	4.59375
30	Cherry (Sour)	2.6125	2.8625	2.19375	2.44375	5.30625	7.625
31	Blueberry *	2.03333	2.05625	2.07083	2.09375	4.15	6.04167
32	Banana *	1.91538	2.375	1.86538	2.325	4.7	8.76923
33	Pomegranate *	1.25	1.3	1.175	1.225	3.425	4.675
34	Plum *	3.62143	4.18214	2.19286	2.75357	6.93571	8.14286
35	Persimmon *	1.51111	1.725	1.54444	1.75833	3.48333	5.16667
36	Prickly Pear	1.66667	1.66667	1.58333	1.58333	3.25	2.65833
37	Sweet Cherry *	3.1381	3.17381	2.55714	2.59286	5.76667	7.61905
38	Grape *	8	8.08333	9.03333	9.11667	17.2	20.1111
39	Pomelo	8.6	8.6	1.9	1.9	10.5	9.62
40	Jackfruit * (Ripe)	4.51429	4.61429	4.37619	4.47619	10.5429	11.2238
41	Blackcurrant	1.66897	1.66897	0.68966	1.56034	4.1	2.65517
42	Gooseberry	2.37209	2.37209	0.44186	0.44186	2.81395	2.37209
43	Dried Peach	1.56098	2.5	1.64634	2.58537	5.08537	7.47561
44	Lychee *	11.5385	11.5385	3.53846	3.53846	11.7154	12.7154
45	Dried Apricot	2.03	2.35	1.22	1.54	1.25	7.25
46	Prune *	3.4	3.41	1.65333	1.66333	5.07333	8.66667
47	Dried Fig	2.7449	3.05612	2.4898	2.80102	4.88776	4.88776
48	Dried Prune	3.82667	3.86	1.97333	2.00667	5	8.66667
49	Raisin (Golden)	9.39394	9.39394	10.5152	10.5152	19.9091	24.2424
50	Raisin	6.93333	6.93333	7.51111	7.51111	14.4444	17.6267
51	Date (Fruit)	5.02985	5.0694	4.77612	4.81567	9.92537	11.194

Note: The data present the approximate carbohydrate content values in various fruits from different public sources. An asterisk (*) represents a distinct group (the most popular fruits) used for analysis.

**Table 3:** Analysis of the individual hexose sugar contents and the GI in various fruits (n = 51).

Values	Glucose	Total Glucose	Fructose	Total Fructose	Total Sugar	Total Carbohydrates
R	0.35	0.36	0.43	0.44	0.29	0.22
R^2^	0.12	0.13	0.19	0.19	0.08	0.05
*p*-values	0.0099	0.0087	0.0013	0.0012	0.0352	0.1072

**Table 4:** Analysis of the individual hexoses-to-dietary fiber ratio and the GI in various fruits (n = 51).

Values	Glucose	Total Glucose	Fructose	Total Fructose	Total Sugar	Total Carbohydrates
R	0.35	0.45	0.50	0.58	0.53	0.57
R^2^	0.12	0.20	0.25	0.33	0.28	0.32
*p*-values	0.01	0.0009	0.0002	<0.0001	<0.0001	<0.0001

**Table 5:** Available carbohydrate content of various fruits, calculated by subtracting dietary fiber from individual hexose sugar contents.

S. No.	Fruits Name	Glucose Minus Fiber	Total Glucose Minus Fiber	Fructose Minus Fiber	Total Fructose Minus Fiber	Total Sugar Minus Fiber	Total Carbohydrate Minus Fiber
1	Avocado *	−6.62	−6.56	−6.62	−6.56	−6.48	1.94
2	Guava *	−7.7	−7.2	−7	−6.5	−4.8	5.4
3	Tomato *	0.05	0.1	0.17	0.22	1.52	2.69
4	Cantaloupe *	0.64	2.815	0.97	3.145	6.86	7.26
5	Watermelon *	0.78	1.385	2.56	3.165	5.35	6.75
6	Grapefruit (Pink)	0.01	1.765	0.17	1.925	5.29	9.1
7	Grapefruit (White)	0.51	2.475	0.67	2.635	6.21	7.31
8	Nectarine	−0.53	1.885	−0.81	1.605	5.69	6.98
9	Pineapple *	0.33	3.325	0.72	3.715	8.44	11.7
10	Raspberry	−4.64	−4.54	−4.15	−4.05	−2.09	5.4
11	Peach *	−1.35	1.03	−1.77	0.61	5.09	6.8
12	Strawberry *	−0.01	0.225	0.44	0.675	2.9	5.68
13	Mango *	−0.89	2.595	1.78	5.265	10.76	12.1
14	Orange *	−0.2	1.9	0.1	2.2	6.5	9.4
15	Blackberry *	−2.99	−2.955	−2.9	−2.865	−0.52	4.31
16	Apricot *	−1.13	1.805	−2.56	0.375	5.68	7.6
17	Dried Apricot	−7.63	−4.695	−9.06	−6.125	−0.82	62.5
18	Apple *	0.03	1.065	3.5	4.535	8	11.4
19	Pear *	−0.62	0.435	2.54	3.595	7.13	13
20	Pear (Bosc)	−0.62	0.435	2.54	3.595	7.13	13
21	Green Apple	0.16	1.125	2.5	3.465	7.09	11.1
22	Goji Berry	−10.16	−9.98	−11.1	−10.92	32.6	64.1
23	Starfruit	0.3	0.7	0.4	0.8	1.18	3.93
24	Red Currant *	−1.08	−0.775	−0.77	−0.465	3.06	9.5
25	Cranberry *	−0.16	−0.08	−2.93	−2.85	0.67	8.4
26	Fig *	0.8	1	−0.1	0.1	13.4	16.3
27	Physalis	−6.8	−5.395	−8.16	−6.755	21.44	50.01
28	Purple Passion	−6.4	−4.75	−7.3	−5.65	0.8	13
29	Kiwi (Fruit) *	0.91	0.985	1.15	1.225	5.41	11.5
30	Cherry (Sour)	2.58	2.98	1.91	2.31	6.89	10.6
31	Blueberry *	2.48	2.535	2.57	2.625	7.56	12.1
32	Banana *	2.38	3.575	2.25	3.445	9.62	20.2
33	Pomegranate *	1	1.2	0.7	0.9	9.7	14.7
34	Plum *	3.67	4.455	1.67	2.455	8.31	10
35	Persimmon *	1.84	2.61	1.96	2.73	8.94	15
36	Prickly pear	2.4	2.4	2.1	2.1	8.1	5.97
37	Sweet Cherry *	4.49	4.565	3.27	3.345	10.01	13.9
38	Grape *	6.3	6.375	7.23	7.305	14.58	17.2
39	Pomelo	7.6	7.6	0.9	0.9	9.5	8.62
40	Jackfruit * (Ripe)	7.38	7.59	7.09	7.3	20.04	21.47
41	Blackcurrant	3.88	8.93	−1.8	3.25	17.98	9.6
42	Gooseberry	5.9	5.9	−2.4	−2.4	7.8	5.9
43	Dried Peach	4.6	12.3	5.3	13	33.5	53.1
44	Lychee *	13.7	13.7	3.3	3.3	13.93	15.23
45	Dried Apricot	10.3	13.5	2.2	5.4	2.5	62.5
46	Prune *	18	18.075	4.9	4.975	30.55	57.5
47	Dried Fig	17.1	20.15	14.6	17.65	38.1	38.1
48	Dried Prune	21.2	21.45	7.3	7.55	30	57.5
49	Raisin (Golden)	27.7	27.7	31.4	31.4	62.4	76.7
50	Raisin	26.7	26.7	29.3	29.3	60.5	74.82
51	Date (Fruit)	27	27.265	25.3	25.565	59.8	68.3

Note: The values provided are approximate estimates of the carbohydrate contents found in various fruits. (*) represents the distinct group of fruits (the most popular fruits) for analysis.

**Table 6:** Analysis of individual sugar content subtracting fiber in various fruits (n = 51).

Values	Glucose	Total Glucose	Fructose	Total Fructose	Total Sugar	Total Carbohydrates
R	0.44	0.45	0.51	0.53	0.335	0.277
R^2^	0.2	0.21	0.26	0.28	0.126	0.077
*p*-values	0.001	0.0007	<0.0001	<0.0001	0.01	0.0486

## Data Availability

The original contributions presented in the study are included in the article/Supplementary Materials; further inquiries can be directed to the corresponding author.

## Supplementary Materials

The following supporting information can be downloaded at https://www.mdpi.com/article/10.3390/foods14040646/s1. Figure S1: The correlation plots between the glycemic index (GI) and carbohydrate content in various fruits (n = 27). Figure S2: The correlation plots between glycemic index (GI) and carbohydrate subtracting fiber in various fruits (n = 29). Figure S3: The correlation plots between GI and net carbohydrates over fiber ratio in various fruits (n = 29). Figure S4: The correlation plots between the GI and sugar content in various fruits (n = 27). Figure S5: The correlation plot demonstrates the relationship between the glycemic load (GL) and sugar content in various fruits (n = 51). Table S1A. Analysis of carbohydrates content and GI in various fruits (n = 27). Table S1B. Analysis of carbohydrates-to-fiber ratio and GI in various fruits (n = 29). Table S2A. Analysis of fruit content subtracting fiber in various fruits (n = 29). Table S2B. Analysis of net content-to-fiber ratio in various fruits (n = 29). Table S3A. Analysis of carbohydrates content and GI in various fruits excluding outliers (n = 44). Table S3B. Analysis focuses on the carbohydrate-to-fiber ratio and GI in various fruits, while excluding outliers (n = 44). Table S4A: Analysis focuses of carbohydrates content and GI in various fruits, while excluding outliers (n = 27). Table S4B. Analyses focus on carbohydrates-to-fiber ratio and GI in various fruits, while excluding outliers (n = 25). Table S5A. Analysis of carbohydrates content and GL in various fruits (n = 51). Table S5B. Analysis of carbohydrate-to-fiber ratio and GL in various fruits (n = 51). Table S6A. Analysis of carbohydrates content and GL in various fruits (n = 29). Table S6B. Analysis of carbohydrates-to-fiber ratio and GL in various fruits (n = 29).

## Abbreviations

The following abbreviations are used in this manuscript:

CGM	continuous glucose monitoring
FGM/isCGM	flash/intermittently scanned continuous glucose monitoring
the GI	glycemic index
GL	glycemic load
HbA1c	hemoglobin A1c
HHS	U.S. Department of Health and Human Services
iAUC	incremental area under the curve
KFCD	Korean Food Composition Database
NAFLD	non-alcoholic fatty liver disease
ROS	reactive oxygen species
RT-CGM	real-time continuous glucose monitoring
SCFAs	short-chain fatty acids
WHO	World Health Organization
