# Supplementary material for: Fruit Carbohydrates and Their Impact on the Glycemic Index: A Study of Key Determinants
Source: Foods. 2025 Feb 14;14(4):646. doi: 10.3390/foods14040646 (PMC11854304; doi:10.3390/foods14040646)
Supplement: Supplementary file 1 [file foods-14-00646-s001.zip › foods-3433522-supplementary.pdf]

Supplementary Materials:

Figure S1: The correlation plots between the glycemic index (GI) and carbohydrate content in various fruits (n=27).

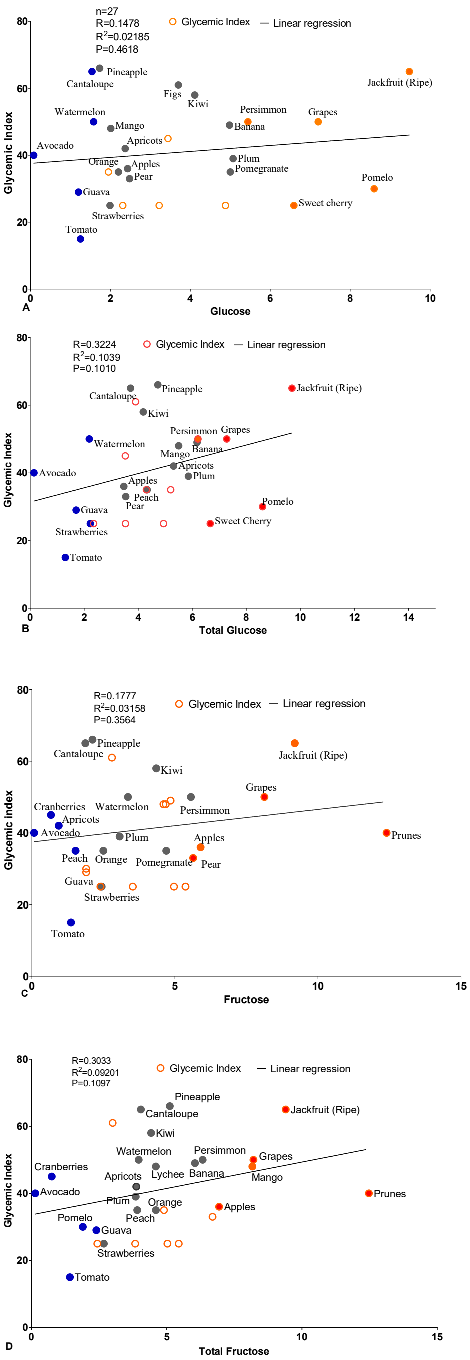

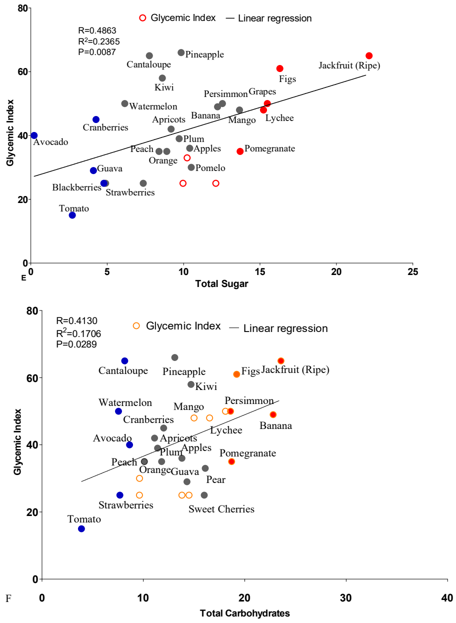

Figure S1. A-F: The correlation plot illustrates the relationship between the glycemic index (GI) and carbohydrate content in various fruits (n=27). The graph depicts the relationship between sugar content and GI, with individual fruit data points plotted. On the Y-axis, GI values are displayed, while the X-axis shows sugar content. The solid line represents the regression curve, indicating the trend between sugar levels and GI across the fruit samples.

Table S1 A. Analysis of carbohydrates content and GI in various fruits (n=27).

Values	Glucose	Total Glucose	Fructose	Total Fructose	Total Sugar	Total Carbohydrates
R	0.1478	0.3224	0.177	0.3033	0.4863	0.413
R²	0.02185	0.1039	0.0315	0.09201	0.2365	0.1706
p-values	0.4618	0.101	0.3564	0.109	0.0087	0.0289

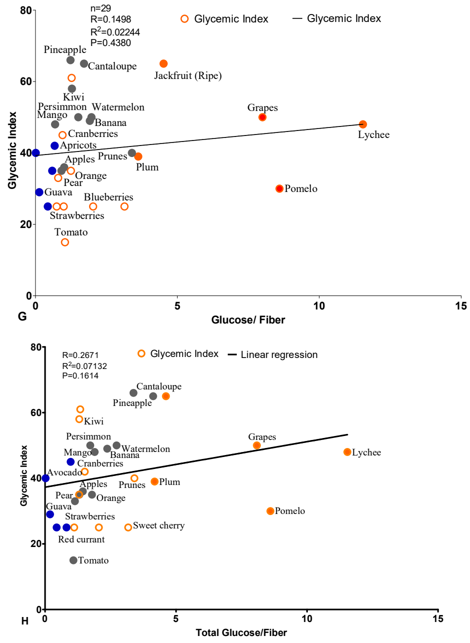

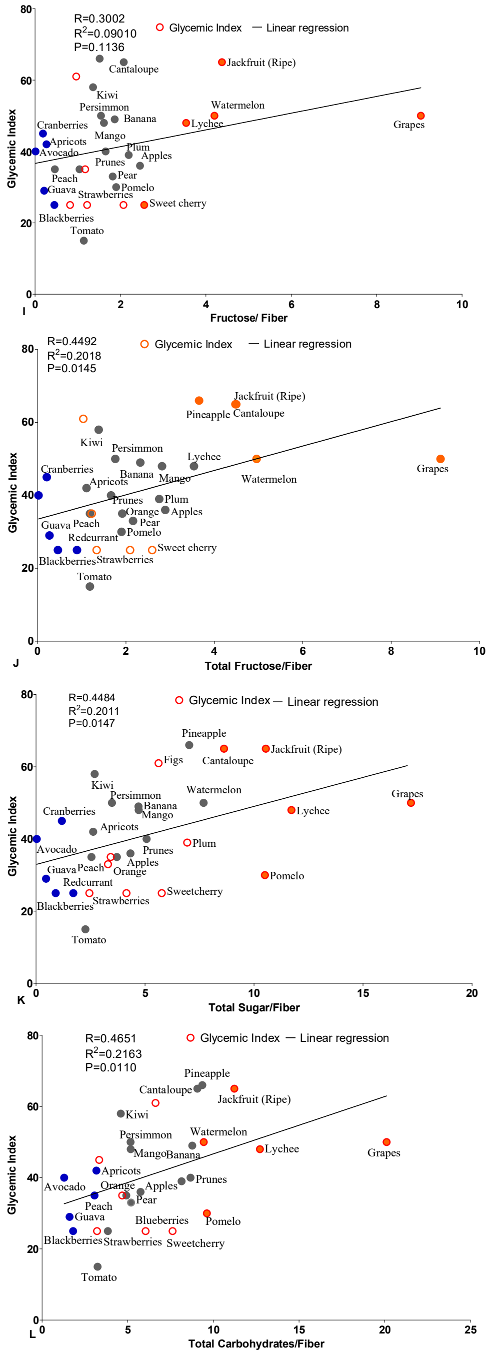

Figure S1 G-L: The correlation plot illustrates the relationship between the GI and the sugar-to-fiber ratio in various fruits (n=29). The graphs depict the correlation between the sugar-to-fiber ratio and GI values. Each data point represents an individual fruit, with GI values plotted on the Y-axis and sugar content on the X-axis. The solid line indicates the regression curve, showing the trend between sugar levels and GI across the fruit samples.

Table S1 B. Analysis of carbohydrates-to-fiber ratio and GI in various fruits (n=29).

**Values**	**Glucose**	**Total Glucose**	**Fructose**	**Total Fructose**	**Total Sugar**	**Total Carbohydrates**
R	0.1498	0.2671	0.3002	0.4492	0.4484	0.465
R^2	0.02244	0.07132	0.0901	0.2018	0.2011	0.216
p-values	0.438	0.1614	0.1136	0.0145	0.0147	0.011

Figure S2: The correlation plots between glycemic index (GI) and carbohydrate subtracting fiber in various fruits (n=29).

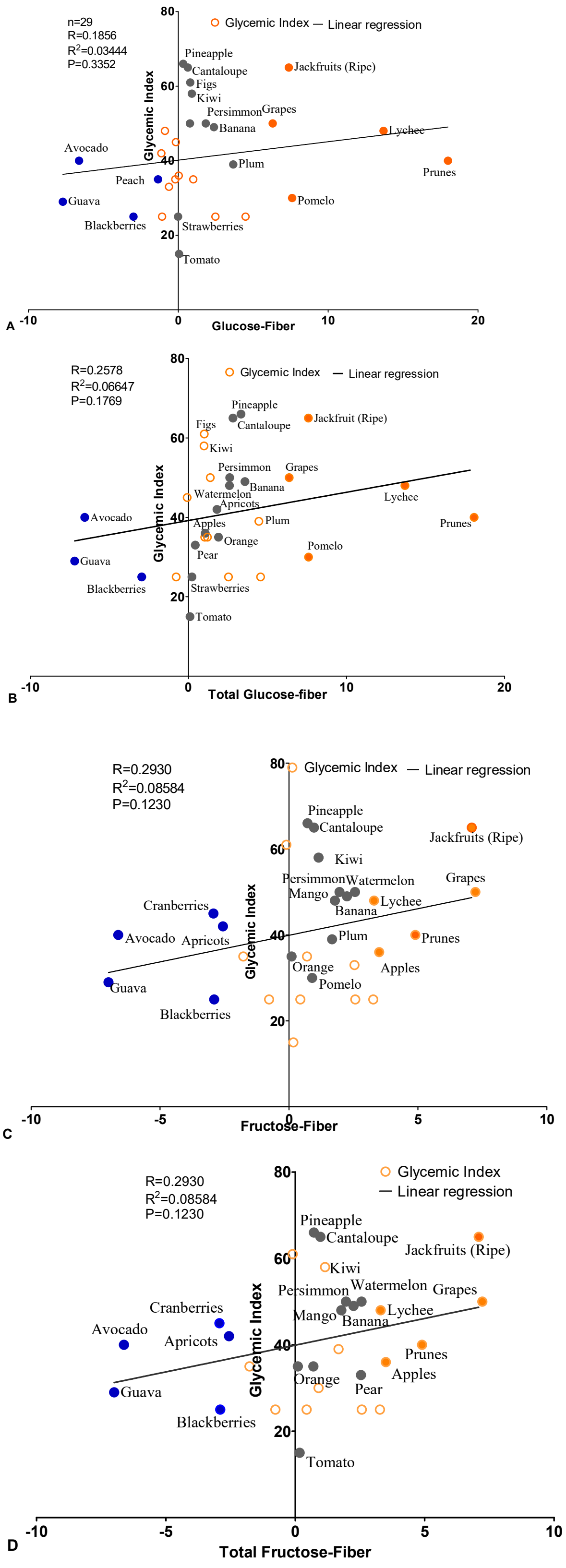

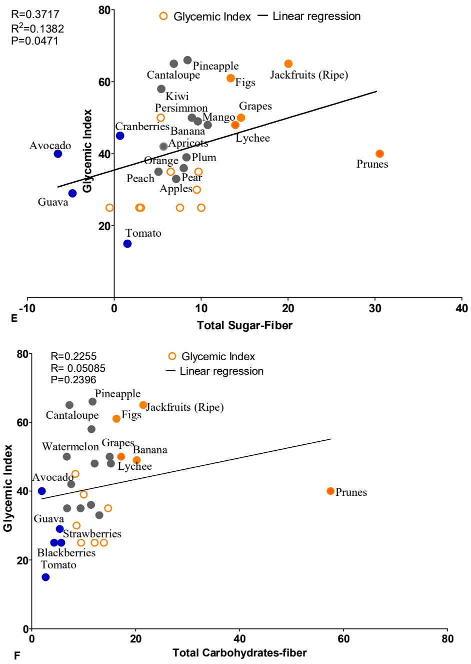

Figure S2 A-F: The correlation plot illustrating the relationship between glycemic index (GI) and carbohydrate subtracting fiber in various fruits (n=29). The graphs depict the correlation between the sugar minus fiber and GI values. Each data point represents an individual fruit, with GI values plotted on the Y-axis and sugar content on the X-axis. The solid line denotes the regression curve, indicating the trend between sugar levels and GI across fruit samples.

Table S2 A. Analysis of fruit content subtracting fiber in various fruits (n=29).

Values	Glucose	Total Glucose	Fructose	Total Fructose	Total Sugar	Total Carbohydrates
R	0.185	0.257	0.29	0.3855	0.3717	0.225
R^2	0.0344	0.06647	0.085	0.1486	0.138	0.05
p-values	0.33	0.176	0.123	0.0389	0.047	0.2396

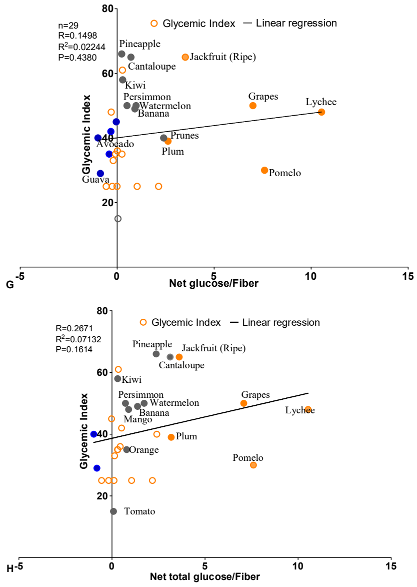

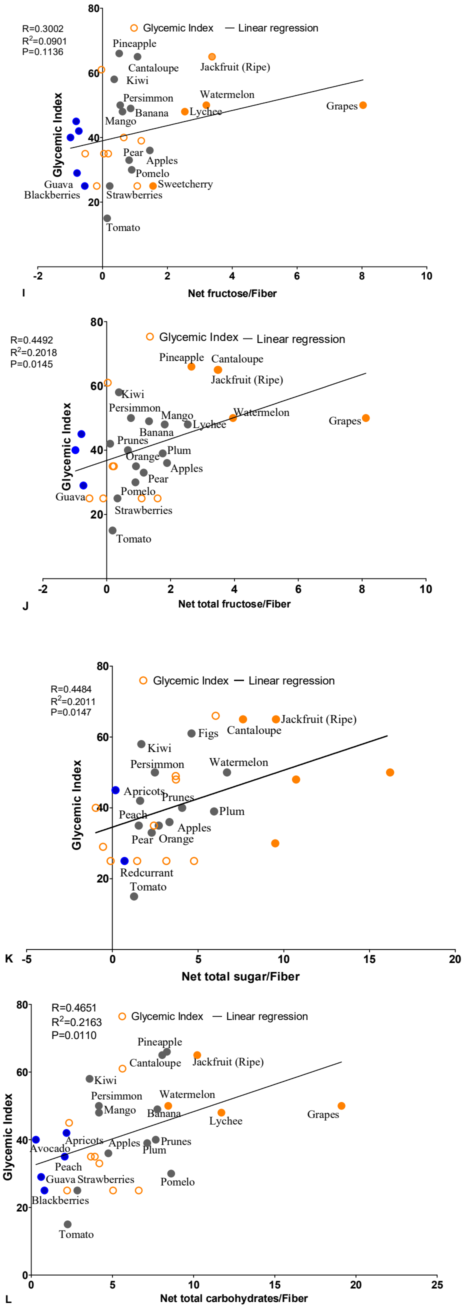

Figure S2 G-L: The correlation plot illustrating the relationship between GI and net carbohydrates over fiber ratio in various fruits (n=29). The graphs depict the correlation between the net carbohydrates over fiber ratio and GI. Each data point represents an individual fruit, with GI values plotted on the Y-axis and net carbohydrates over fiber ratio on the X-axis. The solid line denotes the regression curve, indicating the trend between sugar levels and GI across fruit samples.

Table S2 B. Analysis of net content-to-fiber ratio in various fruits (n=29).

Values	Glucose	Total Glucose	Fructose	Total Fructose	Total Sugar	Total Carbohydrates
R	0.149	0.2671	0.3002	0.4492	0.4484	0.4651
R^2	0.02244	0.07132	0.0901	0.2018	0.2011	0.2163
p-values	0.438	0.1614	0.1136	0.0145	0.0147	0.011

Figure S3: The correlation plots between GI and net carbohydrates over fiber ratio in various fruits (n=29).

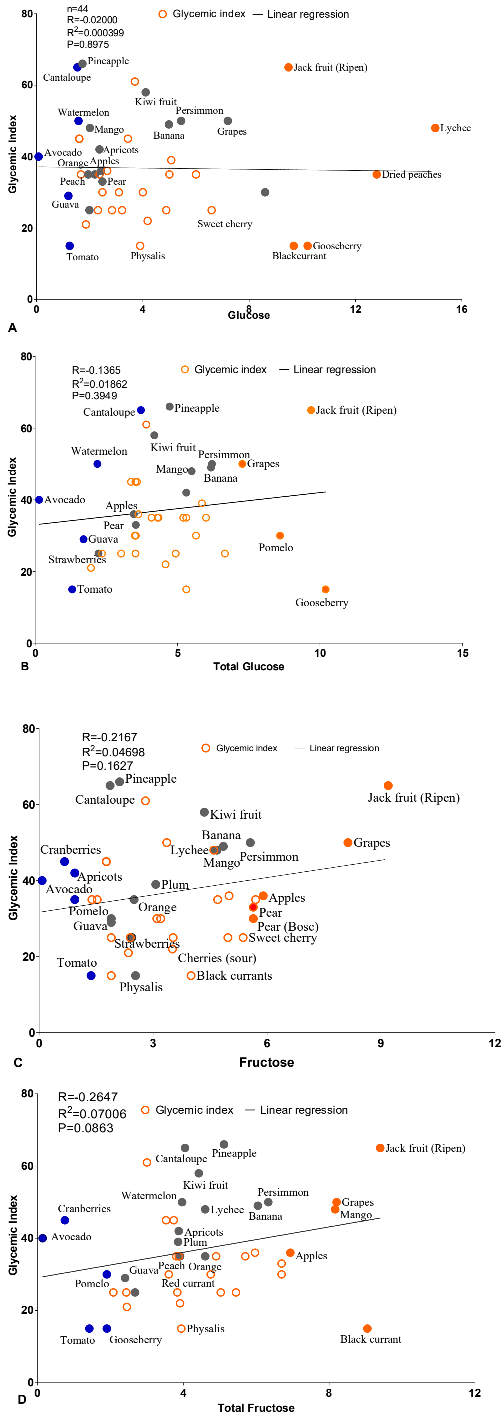

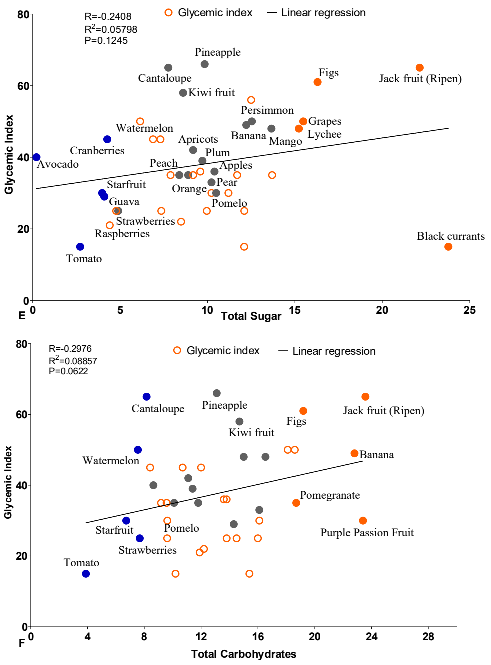

Figure S3 A-F: The correlation plot illustrates the relationship between the GI and sugar content in various fruits, excluding outliers (n=44). The graph shows the correlation between carbohydrates and GI. Each data point represents an individual fruit, with GI values plotted on the Y-axis and carbohydrate content on the X-axis. The solid line represents the regression curve, indicating the trend between sugar levels and GI across the fruit samples.

Table S3 A. Analysis of carbohydrates content and GI in various fruits excluding outliers (n=44).

Values	Glucose	Total Glucose	Fructose	Total Fructose	Total Sugar	Total Carbohydrates
R	-0.02	0.13	0.21	0.26	0.24	0.29
R^2	0.0003	0.018	0.046	0.07	0.05	0.08
p-value	0.89	0.39	0.16	0.08	0.12	0.06

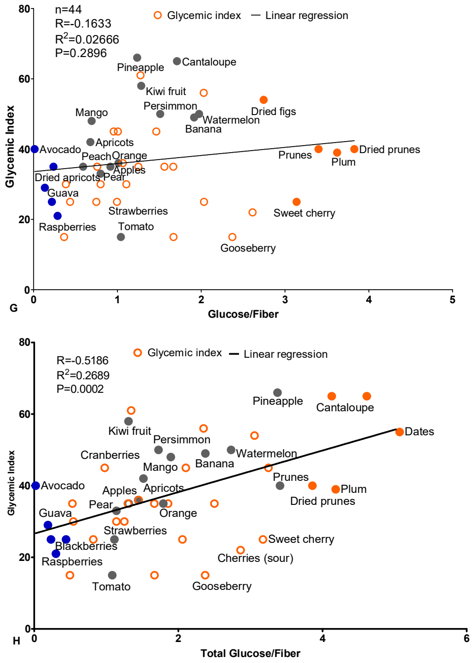

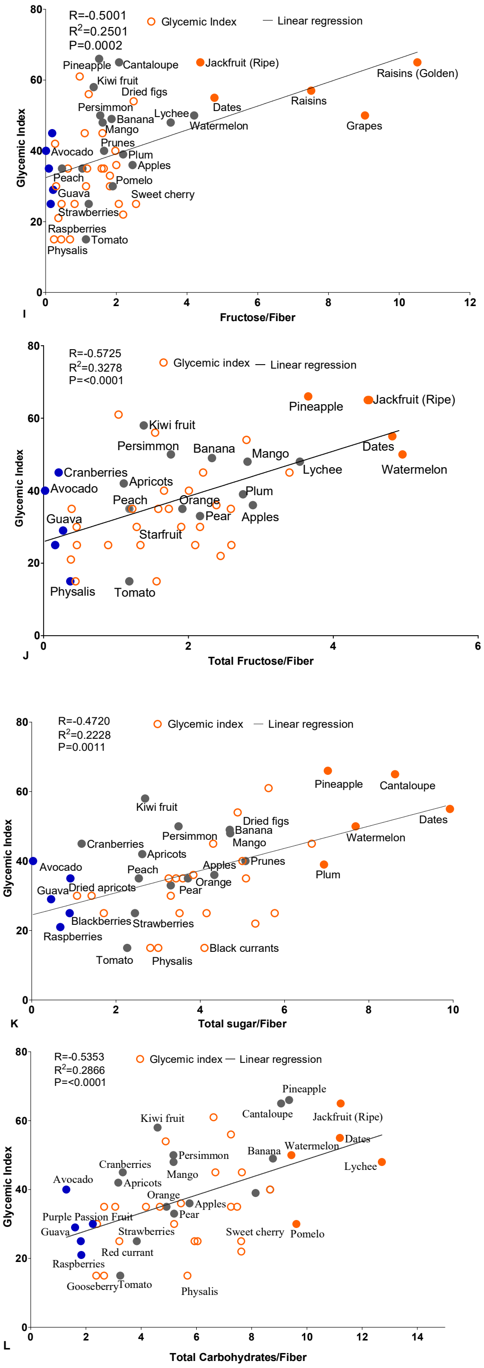

Figure S3 G-L: The correlation plot demonstrates the relationship between GI and carbohydrate content in various fruits, excluding outliers (n=44). The graphs illustrate the correlation between the carbohydrate-to-fiber ratio and GI values. Each data point represents an individual fruit, with GI values plotted on the Y-axis and the carbohydrate-to-fiber ratio on the X-axis. The solid line indicates the regression curve, highlighting the trend between sugar levels and GI across the fruit samples.

Table S3 B. Analysis focuses on the carbohydrate-to-fiber ratio and GI in various fruits, while excluding outliers (n=44).

Values	Glucose	Total Glucose	Fructose	Total Fructose	Total Sugar	Total Carbohydrates
R	0.16	0.51	0.5	0.57	0.47	0.53
R^2	0.02	0.26	0.25	0.32	0.22	0.28
p-value	0.28	0.0002	0.0002	<.0001	0.0011	<.0001

Figure S4: The correlation plots between the GI and sugar content in various fruits (n=27).

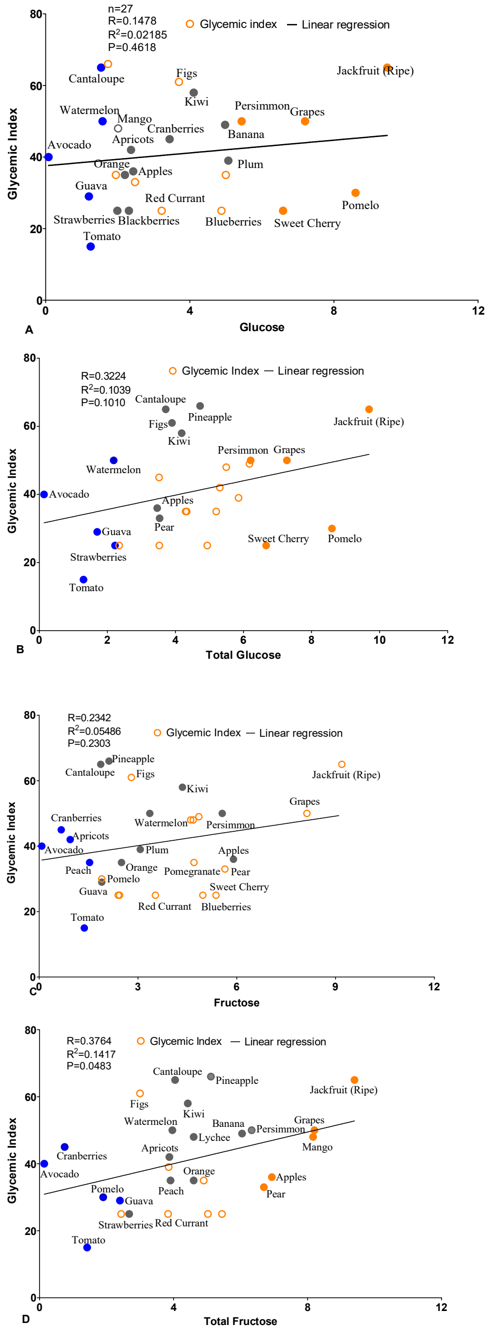

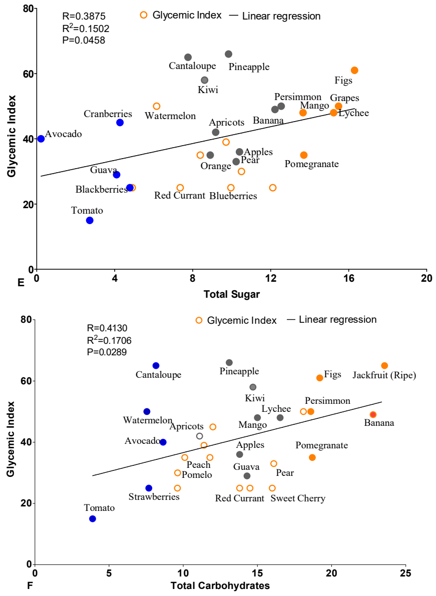

Figure S4 A-F: The correlation plot illustrates the relationship between the GI and sugar content in various fruits (n=27). The graphs depict the relationship between carbohydrate content and GI. Each data point represents an individual fruit, with GI values plotted on the Y-axis and carbohydrate content on the X-axis. The solid line represents the regression curve, indicating the trend between sugar levels and GI across the fruit samples.

Table S4 A: Analysis focuses of carbohydrates content and GI in various fruits, while excluding outliers (n=27).

Values	Glucose	Total Glucose	Fructose	Total Fructose	Total Sugar	Total Carbohydrates
R	0.14	0.32	0.23	0.37	0.38	0.41
R^2	0.02	0.1	0.05	0.141	0.15	0.17
p-value	0.46	0.1	0.23	0.04	0.04	0.02

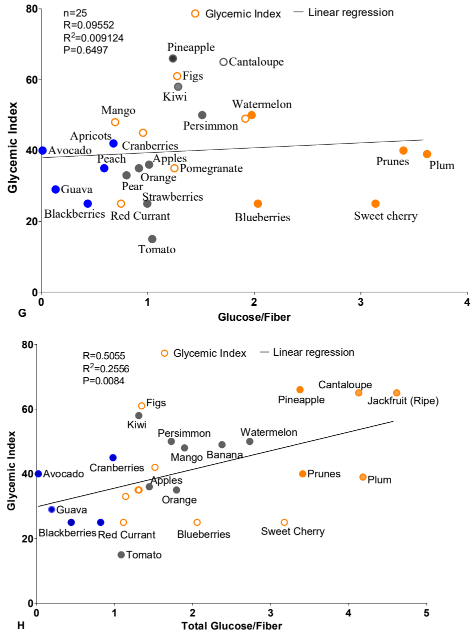

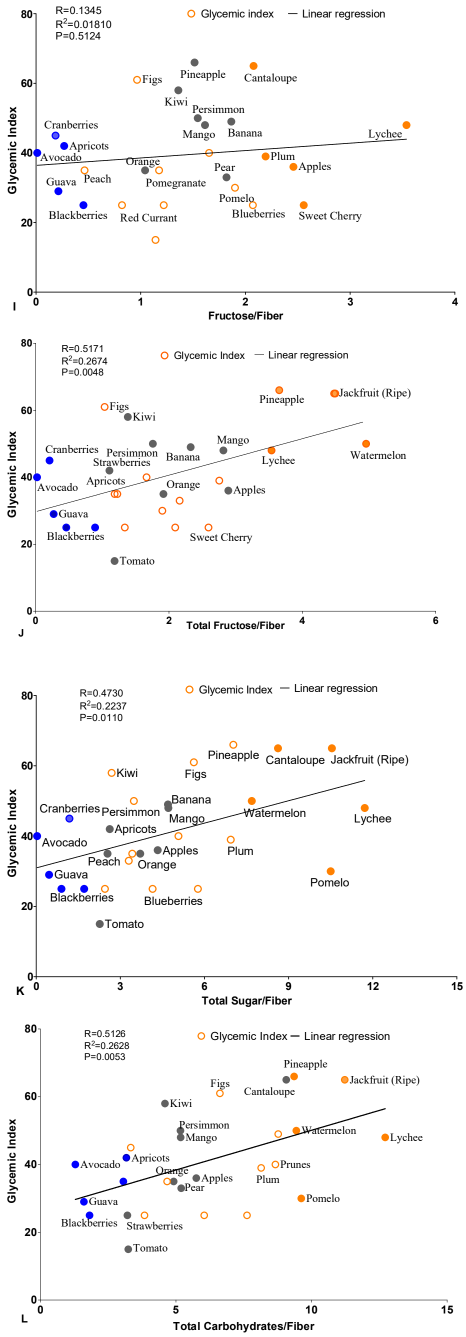

Figure S4 G-L: The correlation plot demonstrates the relationship between the glycemic index (GI) and sugar content in various fruits (n=25). The graphs show the relationship between carbohydrate-to-fiber ratio and GI. Each data point represents a specific fruit, with GI values plotted on the Y-axis and carbohydrate content on the X-axis. The solid line represents the regression curve, illustrating the trend between sugar levels and GI across the fruit samples.

Table S4 B. Analyses focus on carbohydrates-to-fiber ratio and GI in various fruits, while excluding outliers (n=25).

Values	Glucose	Total Glucose	Fructose	Total Fructose	Total Sugar	Total Carbohydrates
R	0.09	0.5	0.13	0.51	0.47	0.51
R^2	0.009	0.25	0.018	0.26	0.22	0.26
p-value	0.64	0.008	0.51	0.0048	0.01	0.0053

Figure S5: The correlation plot demonstrates the relationship between the glycemic load (GL) and sugar content in various fruits (n=51).

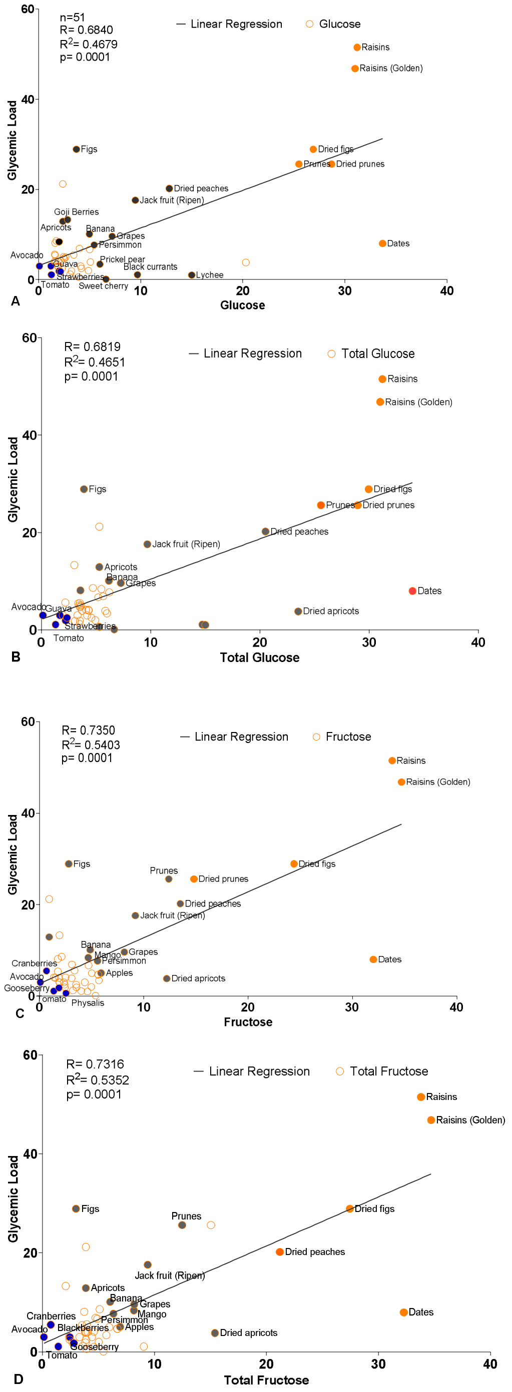

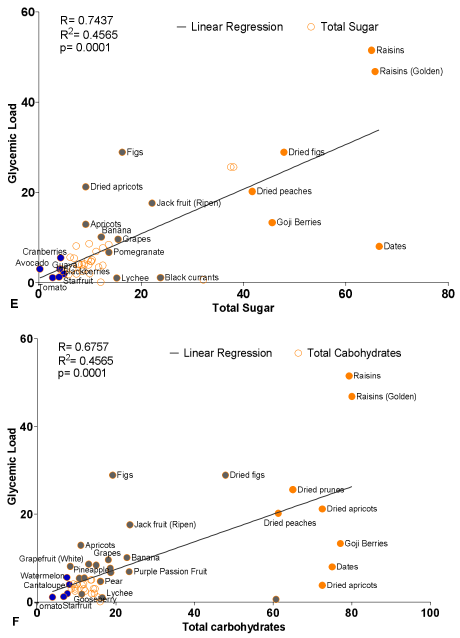

Figure S5 A-F: The correlation plot demonstrates the relationship between the glycemic load (GL) and sugar content in various fruits (n=51). The graphs show the relationship between carbohydrate content and GL. Each data point represents a specific fruit, with GL values plotted on the Y-axis and carbohydrate content on the X-axis. The solid line represents the regression curve, illustrating the trend between sugar levels and GL across the fruit samples.

Table S5 A. Analysis of carbohydrates content and GL in various fruits (n=51).

Values	Glucose	Total Glucose	Fructose	Total Fructose	Total Sugar	Total Carbohydrates
R	0.6840	0.6819	0.7350	0.7316	0.7437	0.6757
R^2	0.4679	0.4651	0.5403	0.5352	0.5530	0.4565
p-values	<0.0001	<0.0001	<0.0001	<0.0001	<0.0001	<0.0001

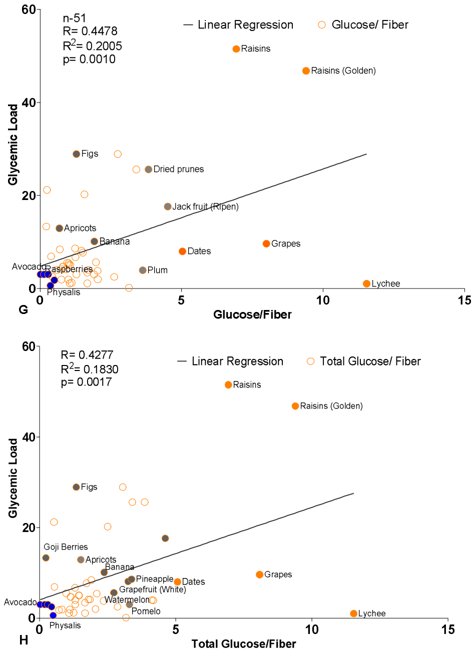

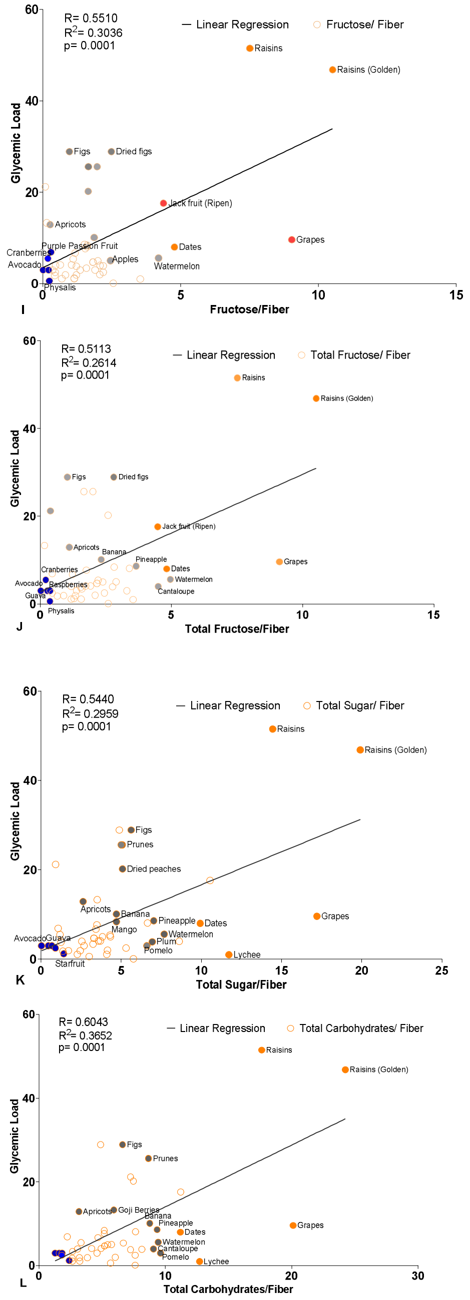

Figure S5 G-L: The correlation plot demonstrates the relationship between the glycemic load (GL) and sugar content in various fruits (n=51). The graphs show the relationship between carbohydrate-to-fiber ratio and GL. Each data point represents a specific fruit, with GL values plotted on the Y-axis and carbohydrate content on the X-axis. The solid line represents the regression curve, illustrating the trend between sugar levels and GL across the fruit samples.

Table S5 B. Analysis of carbohydrate-to-fiber ratio and GL in various fruits (n=51).

Values	Glucose	Total Glucose	Fructose	Total Fructose	Total Sugar	Total Carbohydrates
R	0.4478	0.4277	0.5510	0.5113	0.5440	0.6043
R^2	0.2005	0.1830	0.3036	0.2614	0.2959	0.3652
p-values	0.0010	0.0017	<0.0001	0.0001	<0.0001	<0.0001

Table S6 A. Analysis of carbohydrates content and GL in various fruits (n=29)

**Values**	**Glucose**	**Total Glucose**	**Fructose**	**Total Fructose**	**Total Sugar**	**Total Carbohydrates**
R	0.4493	0.4856	0.4701	0.5001	0.7224	0.6691
R^2	0.2019	0.2358	0.2210	0.2501	0.5219	0.4478
p-values	0.0145	0.0076	0.0101	0.0057	<0.0001	<0.0001

Table S6 B. Analysis of carbohydrates-to-fiber ratio and GL in various fruits (n=29)

**Values**	**Glucose**	**Total Glucose**	**Fructose**	**Total Fructose**	**Total Sugar**	**Total Carbohydrates**
R	0.006923	0.005650	0.09909	0.09236	0.1929	0.2465
R^2	4.793e-005	3.192e-005	0.009818	0.008530	0.03721	0.06074
p-values	0.9716	0.9768	0.6091	0.6337	0.3161	0.1975
